# Supplementary material for: Seminoma and Embryonal Carcinoma Footprints Identified by Analysis of Integrated Genome-Wide Epigenetic and Expression Profiles of Germ Cell Cancer Cell Lines
Source: PLoS One. 2014 Jun 2;9(6):e98330. doi: 10.1371/journal.pone.0098330 (PMC4041891; doi:10.1371/journal.pone.0098330)
Supplement: File S1 — ZIP file containing DMRforPairs output for significant regions. Please start from the html files. (ZIP) [file pone.0098330.s008.zip › figures/4318.pdf]

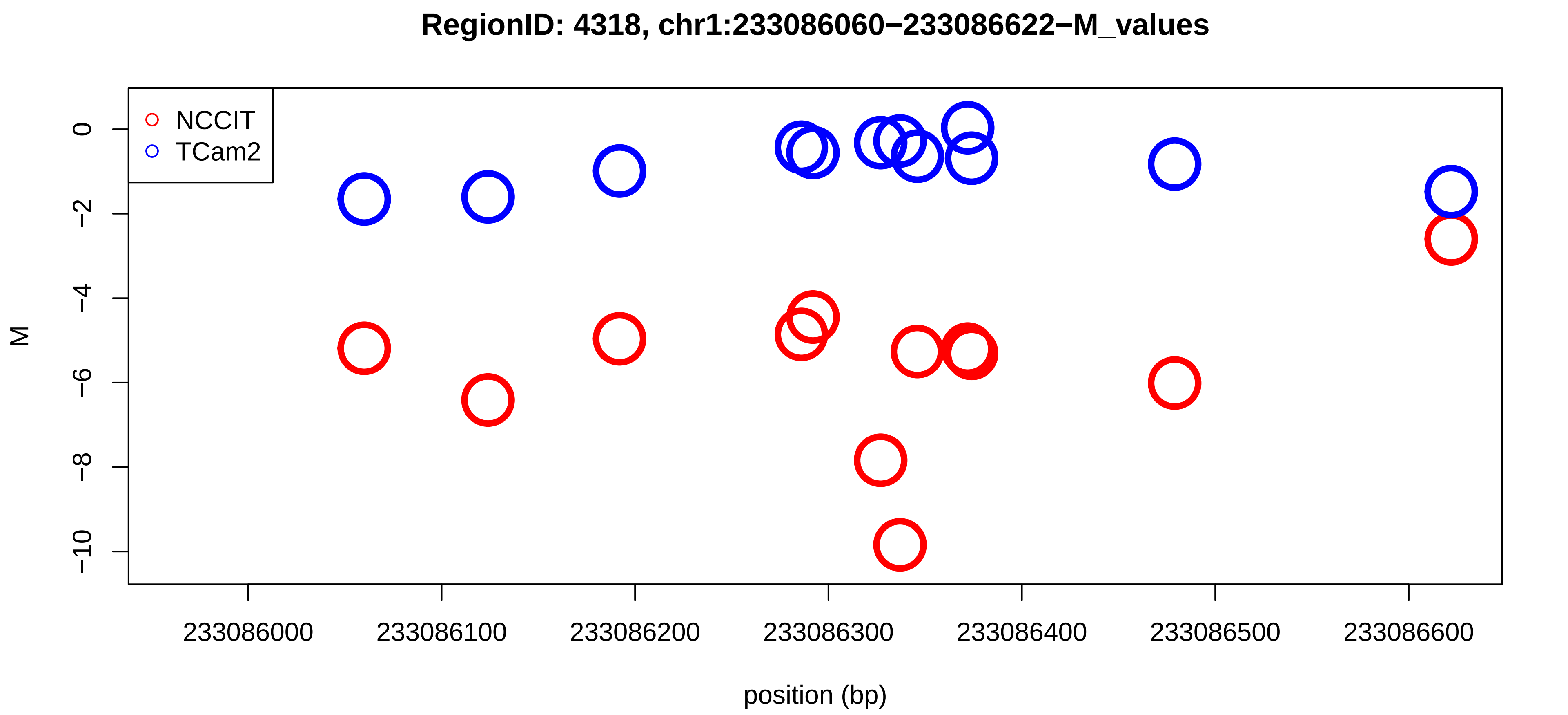
RegionID: 4318, chr1:233086060−233086622−M_values
NCCIT
TCam2
M
0
−2
−4
−6
−8
−10
233086000
233086100
233086200
233086300
233086400
233086500
233086600
position (bp)

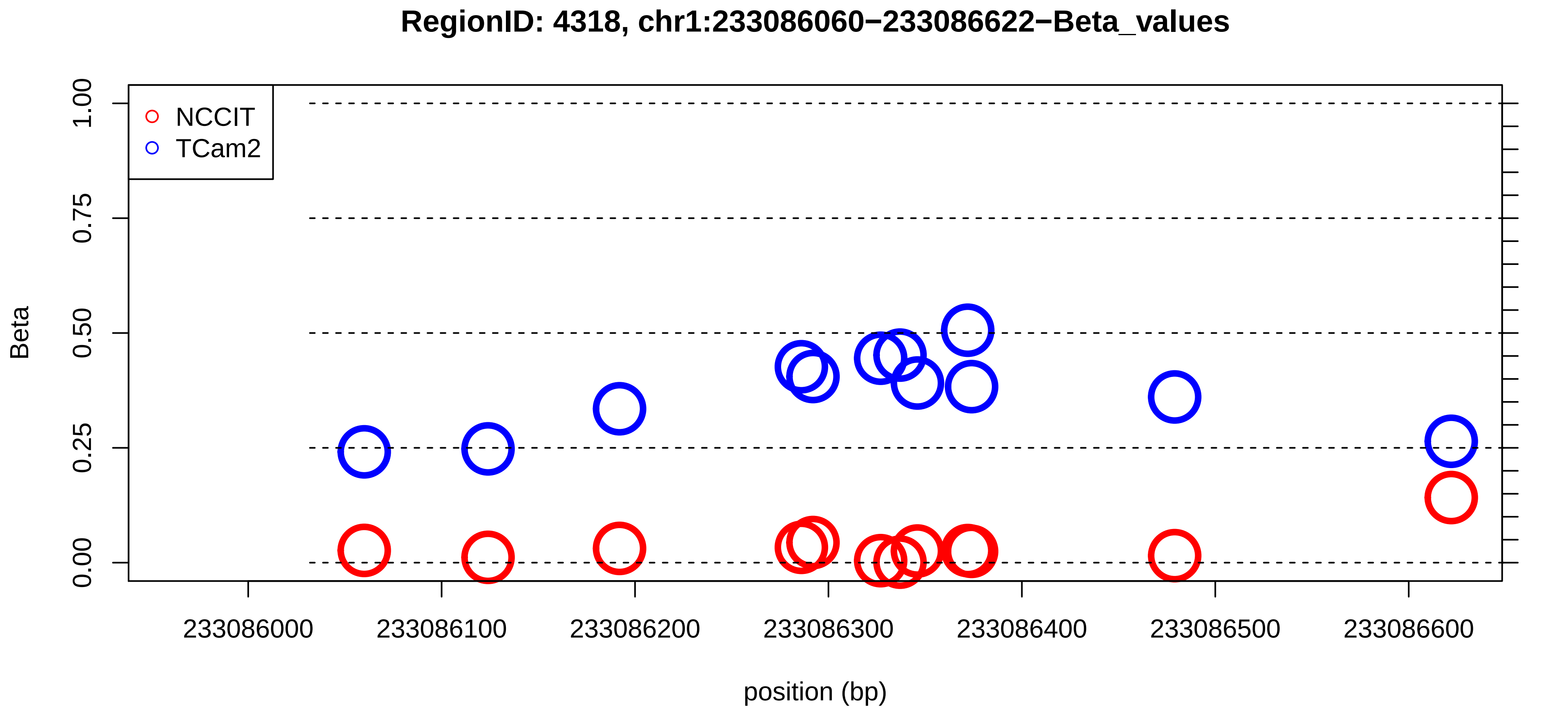
RegionID: 4318, chr1:233086060−233086622−Beta_values
NCCIT
TCam2
Beta
1.00
0.75
0.50
0.25
0.00
233086000
233086100
233086200
233086300
233086400
233086500
233086600
position (bp)

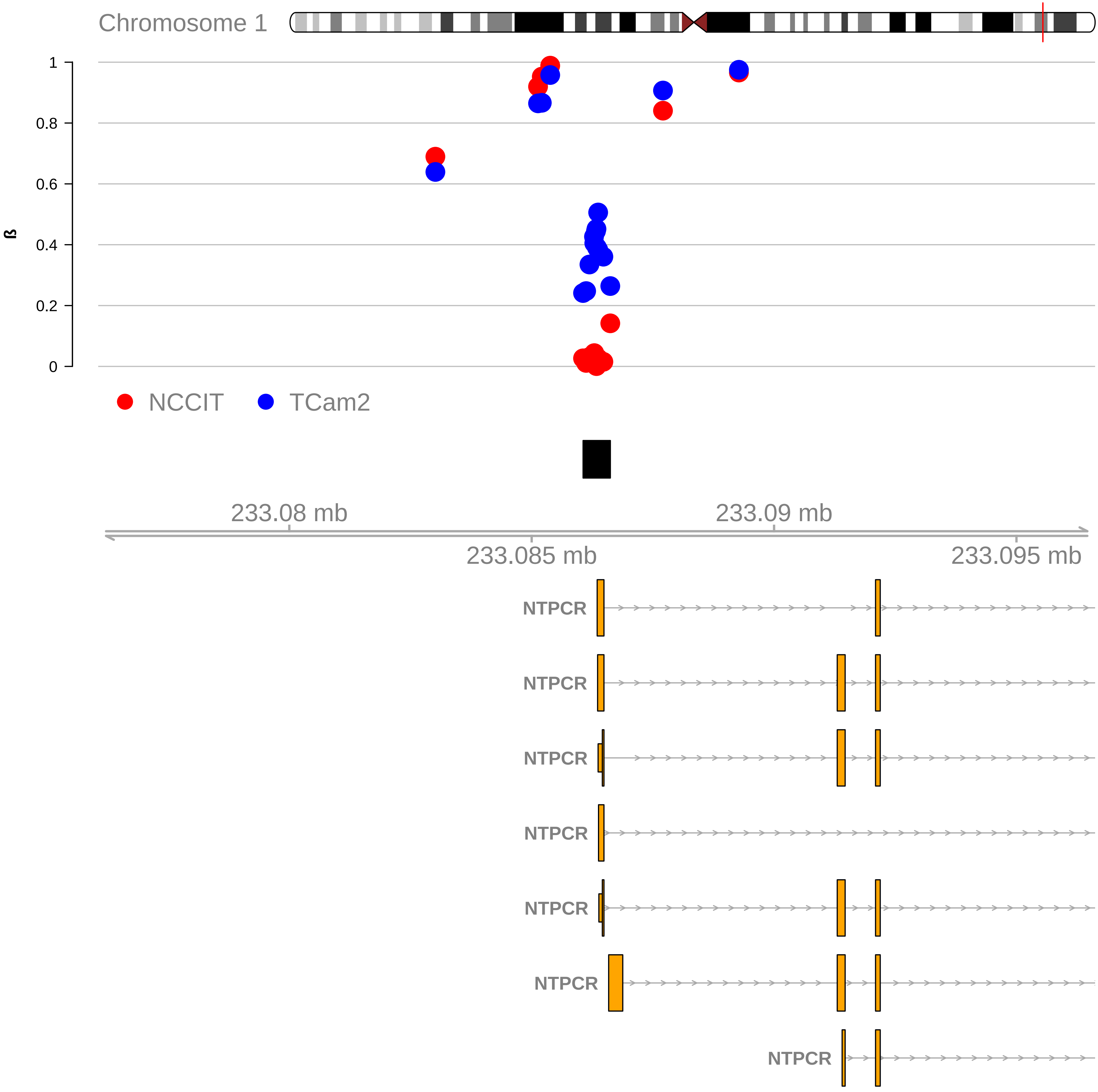
Chromosome 1
ß
1
0.8
0.6
0.4
0.2
0
NCCIT
TCam2
233.08 mb
233.09 mb
233.085 mb
233.095 mb
NTPCR
NTPCR
NTPCR
NTPCR
NTPCR
NTPCR
NTPCR
